# Effect of postoperative application of esketamine on postoperative depression and postoperative analgesia in patients undergoing pancreatoduodenectomy: a randomized controlled trial protocol

**DOI:** 10.1186/s13063-023-07575-8

**Published:** 2023-08-19

**Authors:** Kaili Yu, Zhenguo Song, Bowen Zhang, Qian Pan, Shan Gan, Shaoyong Yang, Quanyong Yang, Xinhua Zuo, Yiqing Yin

**Affiliations:** Department of Anesthesiology, Tianjin Medical University Cancer Institute & Hospital, National Clinical Research Center for Cancer, Tianjin’s Clinical Research Center for Cancer, Key Laboratory of Cancer Prevention and Therapy, Tianjin, China

**Keywords:** Esketamine, Pancreatoduodenectomy, Postoperative depression, Postoperative analgesia

## Abstract

**Background:**

Pancreatoduodenectomy (PD) is traumatic, difficult to perform, and has a high incidence of postoperative complications and perioperative mortality. Postoperative complications and pain occur frequently and seriously affect the psychological status of patients. Esketamine, an N-methyl-D-aspartate (NMDA) receptor antagonist, has analgesic and antidepressant effects. In this study, we aim to investigate the effect of esketamine on postoperative depression and pain in patients undergoing PD.

**Methods/design:**

This prospective, single-center, randomized control trial will include 80 patients who will undergo elective PD. The patients will be randomly assigned to two groups: the experimental group that will receive esketamine (*n* = 40) and the control group (*n* = 40). In the esketamine group, the analgesic pump will be connected immediately after surgery. A solution of esketamine 1.5 mg/kg + sufentanil 2 µg/kg, diluted to 150 mL, will be administered continuously for 72 h at the background infusion and impact doses of 1 mL/h and 2 mL/time, respectively; the locking time will be 10 min. The control group will receive sufentanil 2 µg/kg that will be administered as per the esketamine group. The primary outcome will be the Hamilton Depression Scale (HAMD-17) score on the third day post-surgery (POD3). Secondary study indicators will include (1) visual analog scale (VAS) score and HAMD-17 score prior to surgery, immediately after entering the postanesthesia care unit (PACU) and 1, 2, 3, 4, and 5 days after surgery; (2) Richmond Agitation-Sedation Scale (RASS) score at 1, 2, 3, 4, and 5 days after surgery; (3) consumed doses of sufentanil and esketamine after surgery; (4) postoperative analgesia pump effective press times, rescue analgesia times, and rescue drug dosage, recording the number of rescue analgesia and rescue drug dosage at 6, 24, 48, and 72 h after the patient enters the PACU; (5) postoperative complications and adverse events; (6) postoperative hospital stay; (7) concentrations of brain-derived neurotrophic factor (BDNP), 5-hydroxytryptamine (5-HT), tumor necrosis factor (TNF-α) and interleukin-6, at 1, 3, and, 5 days post-surgery; and (8) the patient survival rate at 6 and 12 months post-surgery.

**Discussion:**

The study hypothesis is that the postoperative HAMD-17 and VAS scores, incidence of postoperative adverse reactions, and concentration of serum markers BDNP, 5-HT, TNF-α, and IL-6 in the experimental group will be lower than those in the control group.

**Trial registration:**

ClinicalTrials.gov ChiCTR2200066303. Registered on November 30, 2022.

Protocol version: 1.0

**Supplementary Information:**

The online version contains supplementary material available at 10.1186/s13063-023-07575-8.

## Background

Pancreatoduodenectomy (PD) is the standard surgical procedure for the treatment of precancerous lesions and some benign conditions in the pancreatic head, distal bile duct, ampulla, and duodenum. It is traumatic, difficult to perform, and has a high incidence of postoperative complications and perioperative mortality. Postoperative complications and pain occur frequently and affect the psychological status of patients. Patients who have undergone PD often experience symptoms of anxiety and depression. Studies have shown that the incidence rates of anxiety and depression in such patients are approximately 33.6 and 27.6%, respectively [1, 2]. Additionally, patients have high postoperative pain scores over a long duration, and their pain is difficult to treat [3]. The untimely treatment of postoperative depression and incomplete analgesia can prolong the hospital stay of patients and increase the incidence of complications and treatment costs, and postoperative pain may aggravate their symptoms of postoperative depression [1]. Therefore, more drugs and accurate models are needed to meet the urgent clinical demands of patients with intractable visceral pain.

The antidepressant effect of esketamine is related to its noncompetitive antagonism of the N-methyl-D-aspartate (NMDA) receptor. The cluster discharge signal of the “anti-reward center” of the lateral habenular nucleus of the brain enhances the inhibition of the “reward center” of the monoamine nuclei in the midbrain downstream of the lateral habenular nucleus, leading to depression [4]. Blocking the cluster discharge of neurons in the lateral habenular nucleus can relieve the excessive inhibition of the “reward center,” playing an antidepressant role [5]. Clinical trials have shown that esketamine can improve short-term depression and pain in patients with cervical cancer after surgery [6, 7]. Furthermore, pain and depression are strongly correlated. Acute and chronic pain are known risk factors for postoperative depression, and perfect postoperative analgesia is a key strategy to reduce the incidence of postoperative depression [8, 9]. Esketamine is DEX-ketamine-isolated and purified from ketamine; it is more powerful and has twice the anesthetic and analgesic effects than racemic ketamine. Ketamine can effectively relieve depression and anxiety [10–12]; thus, the study hypothesizes that the postoperative application of esketamine can effectively improve postoperative depression in patients undergoing PD.

Studies have shown that esketamine has multiple effects, such as antipleasure deficiency, reducing suicidal ideation, relieving anxiety, relieving bronchospasm, improving stress, treating chronic pain, protecting neurons, and improving neural plasticity and neuronal activity [13, 14]. Therefore, in patients undergoing PD, Esketamine could have an antidepressant effect, improve mood as a result of its superior analgesic effect, reduce the incidence of adverse reactions, and increase patient satisfaction. Hence, the aim of this study is to investigate the effect of esketamine on postoperative depression and pain in patients undergoing PD.

## Methods

### Study setting and design

**Fig. 1:**
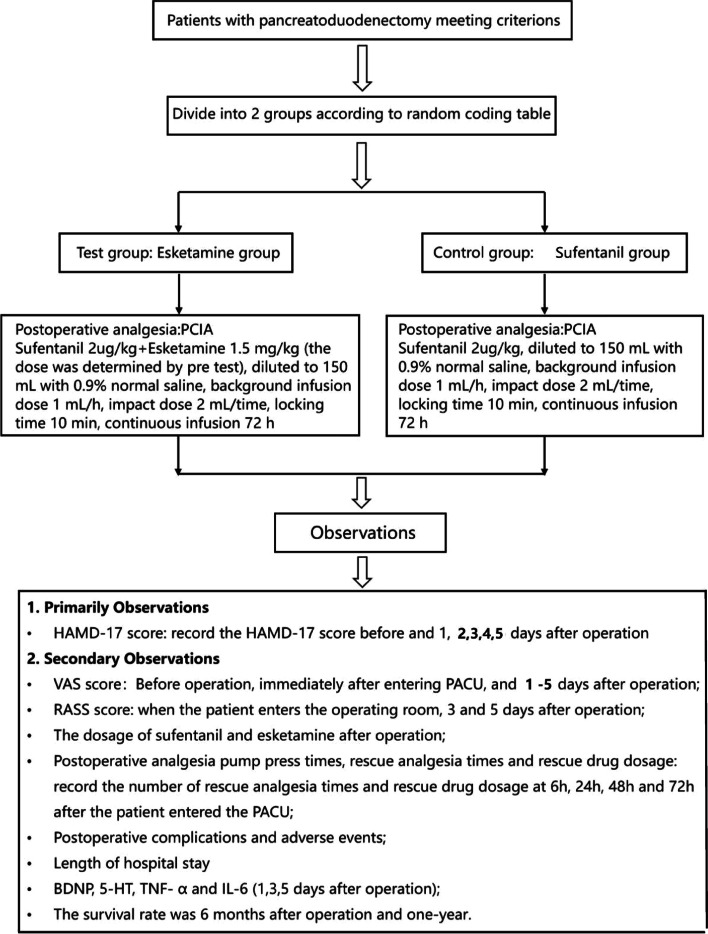
Trial profile. *PCIA*, patient-controlled intravenous analgesia; *HAMD-17*, Hamilton Depression Scale; *VAS*, visual analog scale; *PACU*, postanesthesia care unit; *RASS*, Richmond Agitation-Sedation Scale; *BDNP*, brain-derived neurotrophic factor; *5-HT*, 5-hydroxytryptamine; *TNF-α*, tumor necrosis factor; *IL-6*, interleukin-6

This is an ongoing prospective, double-blind, parallel-group, allocation ratio 1:1, randomized controlled, non-inferiority trial conducted at the Department of Anesthesiology, Tianjin Medical University Cancer Institute and Hospital, Tianjin, China. The trial has been approved by the hospital’s Clinical Research Ethics Committee (E20221148). Written informed consent will be obtained from all patients included in the study. The Trial Steering Group and the independent Data Monitoring and Ethics Committee meet to review conduct throughout the trial period. This study was registered on the Chinese Clinical Trial Register Network (http://www.chictr.org/; registration number, ChiCTR2200066303, 30/11/22). The primary aim of the study is to investigate the effect of postoperative administration of esketamine on postoperative depression in patients undergoing PD. Secondary aims are to investigate the effect of esketamine combined with sufentanil on postoperative analgesia and evaluate the effect of esketamine on the incidence of postoperative adverse events and on the serum markers, brain-derived neurotrophic factor (BDNP), 5-hydroxytryptamine (5-HT), tumor necrosis factor alpha (TNF-α), and interleukin-6 (IL-6). The CONSORT flow diagram is shown in Fig. 1.

### Inclusion criteria

(1) Age: 18–65 years, regardless of sex; (2) American Society of Anesthesiologists (ASA) Class I–III; (3) elective PD to be scheduled; (4) No other surgical contraindications; and (5) can clearly understand and voluntarily participate in the study and sign the informed consent form. If patients are willing to participate, they will be evaluated by Mini-Mental State Examination (MMSE), Hamilton Depression Scale (HAMD-17) [15], and Richmond Agitation Sedation Scale (RASS) [16] before surgery after they sign the informed consent form.

### Exclusion criteria

(1) Opioid analgesics were administered before surgery; (2) drug use or alcohol or drug abuse; (3) patients with preoperative cognitive impairment based on a Mini-Mental Scale Exam scale (normal cut-off value: > 23 points); (4) history of severe cerebrovascular disease; (5) mental illness, patients with a clear diagnosis of depression and long-term drug treatment; (6) no written informed consent for the study and/or no acceptance to have PD surgery; (7) sensitivity to esketamine and related drugs; (8) previous contraindications to ketamine use (e.g., severe refractory hypertension, cor pulmonale, hyperthyroidism, epilepsy, increased intraocular pressure, increased intracranial pressure, intracranial space-occupying lesions, and a history of cerebrovascular accidents); and (9) the investigator thinks that the patient should not participate in this trial.

### Randomization and blinding

All researchers, patients, and analysts will be blinded. Randomization is conducted using a computer-generated blocked randomization sequence. SPSS 21.0 (IBM SPSS, Armonk, NY, USA) will be used to generate a random code table according to the total number of subjects, number of groups, proportion between groups, and length of the block. All participants that meet the inclusion criteria are randomly assigned to either the experimental or control group at a ratio of 1:1, using numbered, sealed, and opaque envelopes. Participants will be grouped and assigned in numerical order. Patient, anesthesia, and postoperative recovery data are written in the case report forms (CRFs). The blind will not be broken during the trial, unless there is significant reason to do so. The outcome will be assented by assessors, and the data will be analyzed by independent statisticians; the outcome evaluation and statistical analysis will be announced independently and blinded. All original records including informed consent and CRFs together with related letters will be retained for 10 years and then destroyed according to the requirements of the hospital.

### Dropout criteria

(1) Patients who voluntarily withdraw their informed consent at any time during the study; (2) failure to complete treatment as planned or refusal of postoperative neuropsychological evaluation; (3) any clinical adverse event or other medical condition in which the continued use of the drug may no longer benefit the subject; (4) lack of main indicators and obvious incomplete data; (5) the efficacy will not be evaluated for cases removed from the group because of adverse reactions, but the adverse effects of treatment should be included in the statistics; and (6) other reasons that the investigators believe may justify the discontinuation of the trial.

### Treatment implementation

To the best of our knowledge, there are no reports in the literature on the dosage of esketamine for postoperative analgesia in patients undergoing PD. Thus, we carried out a preliminary experiment in four groups with five people in each (E20210966A). The doses of the three test groups were 1.0 mg/kg of esketamine + 2 μg/kg of sufentanil; 1.5 mg/kg of esketamine + 2 μg/kg of sufentanil; and 2.0 mg/kg of esketamine + 2 μg/kg of sufentanil, respectively. The control group received 2 μg/kg of sufentanil. These drugs were diluted to 150 mL using 0.9% saline. Patient-controlled intravenous analgesia was used with the background infusion and impact doses of 1 mL/h and 2 mL/time, respectively, administered continuously over 72 h. The locking time was 10 min. The frequencies of postoperative analgesia pump pressing and rescue analgesia were outcome indicators. There were no statistically significant differences in the frequency of analgesic pump presses and number of remedial analgesia between groups; the preliminary experimental results showed that the test group had the best analgesic effect with esketamine 1.5 mg/kg and sufentanil 2 μg/kg (Table 1).

**Table 1:** Experimental results

**Number**	**Gender**	**Age (year)**	**Height (cm)**	**Weight (kg)**	**Other underlying diseases complicated before surgery**	**Preoperative MMSE score**	**Remedy analgesic frequency and medication**	**Number of effective compressions of analgesic pump**	**Total number of remedial analgesia + number of effective compressions of analgesia pump**
**Experimental group 1: esketamine 1.0 mg/kg, sufentanil 2 µg/kg**									
**1**	Male	71	175	80	Hypertension	29	0	20	86
**2**	Female	62	155	50	Hypertension, myocardial ischemia	29	0	18	
**3**	Female	56	162	60		29	0	32	
**4**	Female	58	160	55		29	1, bucinazine 100 mg	5	
**5**	Female	51	150	60		29	0	10	
**Experimental group 2: esketamine 1.5 mg/kg, sufentanil 2 µg/kg**									
**6**	Male	59	164	70	Hypertension	29	0	37	73
**7**	Male	47	170	65		27	1, piperidine 50 mg	22	
**8**	Female	53	164	65		29	0	12	
**9**	Female	43	160	60		29	0	1	
**10**	Female	53	163	79	Hypertension, coronary heart disease	29	0	0	
**Experimental group 3: esketamine 2 mg/kg, sufentanil 2 µg/kg**									
**11**	Male	40	162	62		27	2, bucinazine 200 mg	22	98
**12**	Male	66	168	75	Hypertension, coronary heart disease	28	0	3	
**13**	Male	70	170	80	Hypertension	28	1, bucinazine 100 mg	8	
**14**	Male	41	170	76		28	0	60	
**15**	Male	61	170	60		28	0	2	
**Control group: sufentanil 2 µg/kg**									
**16**	Male	53	172	80	Hypertension, diabetes	29	2, bucinazine 200 mg	20	139
**17**	Female	69	160	60	Hypertension	29	1, bucinazine 100 mg	39	
**18**	Male	62	170	74		29	0	39	
**19**	Female	61	157	70		29	0	36	
**20**	Male	41	173	72		28	1, bucinazine 100 mg	1	

The control group received sufentanil using patient-controlled intravenous analgesia. The dose of sufentanil was 2 μg/kg, diluted to 150 mL with 0.9% normal saline; the background infusion and impact doses were 1 mL/h and 2 mL/time with a locking time of 10 min, respectively, and infused continuously over 72 h.

The test group received esketamine. Patients were immediately connected to the analgesia pump after surgery. Esketamine 1.5 mg/kg + sufentanil 2 μg/kg was diluted to 150 mL using 0.9% normal saline and administered continuously over 72 h, with the background infusion and impact doses of 1 mL/h and 2 mL/time with a locking time of 10 min, respectively. There is no compensation or additional care for the subjects in this study.

### Data collection and follow-up period

The primary outcome measure would be the HAMD-17 score on the third postoperative day. Secondary study indicators include (1) the visual analog scale (VAS) and HAMD-17 scores prior to surgery, immediately after entering the PACU, and 1, 2, 3, 4 and 5 days after operation; (2) RASS score when the patient enters the operating room and at 1, 2, 3, 4 and 5 days after surgery; (3) the dosage of sufentanil and esketamine after surgery; (4) postoperative analgesia pump press times, rescue analgesia times, rescue drug dosage, and number of rescue analgesia times and rescue drug dosages at 6, 24, 48, and 72 h after the patient entered the PACU; (5) postoperative complications and adverse events; (6) postoperative hospital stay; (7) concentrations of brain-derived neurotrophic factor (BDNP), 5-hydroxytryptamine (5-HT), tumor necrosis factor (TNF-α), and interleukin-6 at 1, 3, and 5 days post-surgery; and (8) the patient survival rate at 6 and 12 months post-surgery. The intervention is self-regulated, and the authors are monitoring usage. Nurses in the ward will be responsible for assisting in the follow-up study of medication to ensure patient compliance. Implementing the esketamine or control group will not require alteration to usual care pathways (including the use of any medication), and these will continue for both trial arms.

Safety end-point measures will include incidence, severity, and causality of reported serious adverse events (SAEs), namely changes in the occurrence of the expected common prematurity complications and clinical laboratory test assessments, and the development of unexpected SAEs in this high-risk population. All SAEs will be followed until a satisfactory resolution or until the investigator responsible for the care of the participant deems the event to be chronic or the patient to be stable. All expected and unexpected SAEs, whether or not they are attributable to the study intervention, will be reviewed by the principal investigator and all authors to determine if there is a reasonable suspected causal relationship to the intervention. If the relationship is reasonable, SAEs will be reported to the Ethics Committee to guarantee the safety of participants.

### Sample size calculation

The clinically meaningful beneficial effect of esketamine on the depressive symptoms of patients undergoing surgery is unclear. The minimum clinically important difference was at least 20%; however, differences in response rates range from approximately 26 to 56% among patients with major depressive disorder [17]. Therefore, we assumed that for surgical patients, the third postoperative day response rate difference would be 30% between the two groups. Likewise, for the control group, a response rate of 10% was assumed. A total of 38 patients in each group would provide 80% power with a two-sided type-I error of 0.05. The test-group-to-control-group ratio is 1. Considering an overall withdrawal rate of 5%, a sample size of 80 patients is considered adequate [10]. If data are missing, we will use multiple imputation methods to fill in according to the research needs.

### Statistical methods

Statistical analysis will be carried out using the “intention to treat” method; all data will be collected in a single database and analyzed to evaluate any differences between the randomized groups for both primary and secondary outcomes. SAS 9.4 (SAS Institute, Cary, NC, USA) statistical software will be used for all statistical analysis. All statistical tests are two-tailed. *P*-values ≤ 0.05 will be considered statistically significant. Normally distributed data will be described as mean ± standard deviation, and the analysis of variance will be used for comparison between groups. Non-normally distributed data will be described as median (minimum–maximum), and the rank-sum test will be used for comparison between groups. The counting data are statistically described by frequency (composition ratio), and the *χ*^2^ test or exact probability method will be used for comparison between groups. The rank-sum test will be used for the inter-group comparison of grade data.

## Discussion

The study hypothesizes that the postoperative HAMD-17 and VAS scores, incidence of postoperative adverse reactions, and concentration of the serum markers BDNP, 5-HT, TNF α, and IL-6 will be lower than those in the control group. The HAMD-17, which was developed by Hamilton in 1960 [18], is the most used scale in the clinical evaluation of depression and can accurately assess the depression status of adult patients [15, 19]. Pain is an independent risk factor for depression [8, 9]. The analgesic effect of esketamine and the effect of treating depression can effectively reduce the postoperative VAS and HAMD-17 scores of patients.

Ketamine is an NMDA receptor antagonist, a widely used anesthetic [20] for the treatment of depression [21]. The most important brain regions associated with depression are the amygdala and hippocampus [22]. The amygdala converts negative information (e.g., anxiety and depression) into neural signals in the brain and is closely related to behavior and mood. Alterations in hippocampal architecture and synaptic remodeling are strongly related to the onset of depression and the effects of antidepressants. Depression is usually caused by reduced levels of monoamine neurotransmitters in the brain, such as serotonin (5-HT). 5-HT is an important neurotransmitter in the brain, with levels closely related to the pathogenesis of depression [23]. BDNF is the most abundant neurotrophic factor in the body, and its association with depression has also recently been confirmed [24, 25]. Moreover, BDNF polymorphisms are associated with the antidepressant efficacy of ketamine in depressed patients [24]. TNF-α and IL-6 are inflammatory indicators and are strongly correlated with postoperative depression [26].

The NMDA receptors are closely related to the development of central sensitization in the dorsal horn neurons [27, 28], which are responsible for transmitting pain signals. Central sensitization occurs with persistent nociceptive inputs caused by inflammation or injury, leading to persistent and gradually exacerbated pain, hyperalgesia, abnormal pain, relative unresponsiveness to opioids in chronic pain, and severe visceral pain [29]. Binding of ketamine to NMDA receptors may reverse these phenomena. Acute and chronic pain are known risk factors for the onset of postoperative depression [30]. Therefore, well-established postoperative analgesia is a key strategy to reduce the incidence of postoperative depression. The follow-up period of this experiment is long and there is a high risk of loss of follow-up. Long-term and effective communication between researchers, patients, and their families is the key to solving this difficulty.

## Trial status

Recruitment began on January 1, 2023. Recruitment is anticipated to end in December 2023. Recruitment for the study is currently ongoing.

### Supplementary Information

**Additional file 1.** SPIRIT 2013 checklist: recommended items to address in a clinical trial protocol and related documents.

## Data Availability

The data collected during the research process will be strictly confidential and only members of the experimental team can access it. Participants will be assigned a trial identification number, and their detailed information will be stored in a secure database. We will request consent for review of participants’ medical records, and for the collection of blood samples to assess concentrations of brain-derived neurotrophic factor (BDNP), 5-hydroxytryptamine (5-HT), tumor necrosis factor (TNF-α) and interleukin-6, at 1, 3, and, 5 days post-surgery. The datasets analyzed during the current study and statistical code are available from the corresponding author on reasonable request, as is the full protocol.

## Abbreviations

PD: Pancreatoduodenectomy
NMDA: N-methyl-D-aspartate
HAMD-17: Hamilton Depression Scale
POD3: Third day post-surgery
VAS: Visual analog scale
RASS: Richmond Agitation-Sedation Scale
PACU: Postanesthesia care unit
BDNP: Concentrations of brain-derived neurotrophic factor
5-HT: 5-Hydroxytryptamine
TNF-α: Tumor necrosis factor
IL-6: Interleukin-6
ASA: American Society of Anesthesiologists
CRF: Case report forms

## References

[1] Kang MJ (2022). Prevalence of psychological symptoms in patients undergoing pancreatoduodenectomy and results of a distress management system: a clinic-based study. Cancer Res Treat.

[2] Disner SG (2011). Neural mechanisms of the cognitive model of depression. Nat Rev Neurosci.

[3] Tung S (2019). Population-level symptom assessment following pancreaticoduodenectomy for adenocarcinoma. JAMA Surg.

[4] Krystal JH (2019). Ketamine: a paradigm shift for depression research and treatment. Neuron.

[5] Vieira F (2021). Ketamine and Esketamine augmentation for suicidal ideation: a randomized, double-blinded clinical trial. Gen Hosp Psychiatry.

[6] Wang J (2020). Use of various doses of s-ketamine in treatment of depression and pain in cervical carcinoma patients with mild/moderate depression after laparoscopic total hysterectomy. Med Sci Monit.

[7] Wang J (2019). Pharmacokinetics and safety of esketamine in chinese patients undergoing painless gastroscopy in comparison with ketamine: a randomized, open-label clinical study. Drug Des Devel Ther.

[8] Campos AI (2022). Genetic risk for chronic pain is associated with lower antidepressant effectiveness: converging evidence for a depression subtype. Aust N Z J Psychiatry.

[9] Krause JS (2017). Clark, Pain intensity, interference, and medication use after spinal cord injury: association with risk of mortality after controlling for socioeconomic and other health factors. Arch Phys Med Rehabil.

[10] Zhou Y (2021). Ketamine alleviates depressive symptoms in patients undergoing intracranial tumor resection: a randomized controlled trial. Anesth Analg.

[11] Liu P (2021). Effect of pretreatment of S-ketamine on postoperative depression for breast cancer patients. J Invest Surg.

[12] Han Y (2022). S-ketamine as an adjuvant in patient-controlled intravenous analgesia for preventing postpartum depression: a randomized controlled trial. BMC Anesthesiol.

[13] Qiu D (2022). Effect of intraoperative esketamine infusion on postoperative sleep disturbance after gynecological laparoscopy: a randomized clinical trial. JAMA Netw Open.

[14] Singh JB (2016). Intravenous esketamine in adult treatment-resistant depression: a double-blind, double-randomization, placebo-controlled study. Biol Psychiatry.

[15] Deligiannidis KM (2021). Effect of zuranolone vs placebo in postpartum depression: a randomized clinical trial. JAMA Psychiat.

[16] Ely EW (2003). Monitoring sedation status over time in ICU patients: reliability and validity of the Richmond Agitation-Sedation Scale (RASS). JAMA.

[17] Kishimoto T (2016). Single-dose infusion ketamine and non-ketamine N-methyl-d-aspartate receptor antagonists for unipolar and bipolar depression: a meta-analysis of efficacy, safety and time trajectories. Psychol Med.

[18] Gukasyan N (2022). Efficacy and safety of psilocybin-assisted treatment for major depressive disorder: Prospective 12-month follow-up. J Psychopharmacol.

[19] Husain MI (2020). Minocycline and celecoxib as adjunctive treatments for bipolar depression: a multicentre, factorial design randomised controlled trial. Lancet Psychiatry.

[20] Culp C (2020). Ketamine use for cancer and chronic pain management. Front Pharmacol.

[21] Smith-Apeldoorn SY (2022). Maintenance ketamine treatment for depression: a systematic review of efficacy, safety, and tolerability. Lancet Psychiatry.

[22] Park H (2022). Attenuated interoceptive processing in individuals with major depressive disorder and high repetitive negative thinking. J Psychiatr Res.

[23] Yamanaka H (2014). A possible mechanism of the nucleus accumbens and ventral pallidum 5-HT1B receptors underlying the antidepressant action of ketamine: a PET study with macaques. Transl Psychiatry.

[24] Laje G (2012). Brain-derived neurotrophic factor Val66Met polymorphism and antidepressant efficacy of ketamine in depressed patients. Biol Psychiatry.

[25] Lepack AE (2014). BDNF release is required for the behavioral actions of ketamine. Int J Neuropsychopharmacol.

[26] Liu H (2022). TNF-α, IL-6 and hsCRP in patients with melancholic, atypical and anxius depression: an antibody array analysis related to somatic symptoms. Gen Psychiatr.

[27] Zeilhofer HU (1992). Differential effects of ketamine enantiomers on NMDA receptor currents in cultured neurons. Eur J Pharmacol.

[28] American Society of Anesthesiologists Task Force on Acute Pain Management (2012). Practice guidelines for acute pain management in the perioperative setting: an updated report by the American Society of Anesthesiologists Task Force on Acute Pain Management. Anesthesiology.

[29] Tosounidis TH (2015). Pain relief management following proximal femoral fractures: options, issues and controversies. Injury.

[30] Quibell R (2011). Ketamine*. J Pain Symptom Manage.

